# Reverse Periodization for Improving Sports Performance: A Systematic Review

**DOI:** 10.1186/s40798-022-00445-8

**Published:** 2022-04-21

**Authors:** José M. González-Ravé, Fernando González-Mohino, Víctor Rodrigo-Carranza, David B. Pyne

**Affiliations:** 1 Sports Training Laboratory, Faculty of Sports Sciences, University of Castilla-La Mancha, Carlos III Avenue, 45008 Toledo, Spain; 2 Facultad de Ciencias de la Vida y de la Naturaleza, Universidad Nebrija, Madrid, Spain; 3 Faculty of Health, Research Institute for Sport and Exercise, University of Canberra, Canberra, ACT Australia

**Keywords:** Training, Athlete development, Adaptation, Model

## Abstract

**Background:**

Reverse periodization is commonly touted as a salient planning strategy to improve sport performance in athletes, but benefits have not been clearly described.

**Objectives:**

We sought to identify the main characteristics of reverse periodization, and the influence of training volume and periodization models on enhancing physiological measures and sports performance.

**Design:**

Systematic review.

**Methods:**

The electronic databases Scopus, PubMed and Web of Science were searched using a comprehensive list of relevant terms.

**Results:**

A total of 925 studies were identified, and after removal of duplicates and studies based on title and abstract screening, 17 studies remained, and 11 finally included in the systematic review. There was a total of 200 athletes in the included studies. Reverse periodization does not provide superior performance improvements in swimming, running, muscular endurance, maximum strength, or maximal oxygen uptake, compared to traditional or block periodization. The quality of evidence levels for the reverse periodization studies was 1b (individual randomized controlled trial) for two investigations, 2b (individual cohort study) for the remaining studies and a mean of 4.9 points in the PEDro scale (range 0–7).

**Conclusions:**

It appears that reverse periodization is no more effective than other forms of periodization in improving sports performance. More comparative studies on this alternative version of periodization are required to verify its effectiveness and utility across a range of endurance sports.

## Key Points

- Reverse periodization is no more effective than other forms of periodization in improving sports performance, muscular endurance, maximum strength, or maximal oxygen uptake.
- The use of reverse periodization likely induces similar improvements to a traditional model in shorter events such as the 100-m swimming event.
- More comparative studies of periodization models in endurance sports require careful planning of experimental design, longer study periods, and where appropriate matching of training volumes and intensities.

## Introduction

Periodization is a process that serves as the macromanagement of an athlete’s training program in the context of the annual plan [1, 2]. Matveyev’s original model of periodization was developed through monitoring of Soviet athletes preparing for the 1952 and 1956 Olympic Games [3]. Periodization continues to be a valid and reliable model for athletes and is the predominant training methodology used in individual sports such as swimming [4–6]. However, prior to Matveyev’s seminal contribution to the topic, there was foundational work that underpins the theory of periodization [3, 7–9]. A large number of authors have conceptualized periodized training in various models, with different variations of the underlying training process, planning, progressions in training volume and intensity, and recovery [10–12]. The original concept of periodization was proposed initially by Boris Kotov in his book “Olympic Sport” in 1916; later, Pihkala [13] postulated a number of principles such as dividing the annual cycle into preparatory, spring and summer phases, and active rest ending the season [14]. These authors have conceptualized various approaches without an accepted formal definition of periodization as promulgated by Kataoka et al. [1]. The term Periodization was originally employed to describe programs taking the form of predetermined sequential chains of specifically focused training periods. Periodization is a cyclical method of training, where the removal of linearity, and appropriate variation in the form of repeating load oscillations, can provide a superior method of training as Stone et al. identify in their recent (and provocative) narrative review [11]. Kiely [12] asserts the term periodization is frequently engaged to describe any form of training plan, regardless of structure. The challenge is to provide evidence-based guidelines on periodization that meet the conceptual and practical requirements of a wide variety of sports and events.

The rationale of periodized models of strength and power training in athletes originated in western countries centering on the work of Stone and O´Bryant [15], Stone and O’Bryant [16] and Fleck [17]. The models from Verkhoshansky or Bondarchuck have become known in Europe for their translations to different European languages such as Italian [18], Spanish [19], German [20] and also English. It soon became apparent that coaches and athletes needed to examine different periodized models other than traditional strength/power approaches. Subsequently, the meta-analysis of Rhea and Alderman [21] concluded that strength training periodization is more effective than non-periodized models for men and women. This conclusion was based on comparing different programming strategies after controlling the different parameters of workload (i.e., volume, intensity, frequency). Similar outcomes were evident in the review of Hartman et al. [22] who evaluated the effects of different short-term periodization models on strength and speed–strength training, with subjects of different performance levels and sports, who used a particular periodization model during the off-season, pre-season and/or in-season conditioning. From the early works of Matveyev [23], based on the general concept of periodized training proposed in the 1960s, the strength–speed model has been adopted by many generations of analysts and coaches [10, 24].

Over recent decades, many approaches have evolved that can be broadly categorized as traditional, block, or reverse periodization, each offering a differing rationale and template for subdivision of the training program into sequential elements. Bompa [25] classified the periodization in mono-,bi-, and tri-cycle with different models from different authors on each: Matveyev Ozolin, Bondarchuck, Tschiene [5]. Stone et al. [11] contend that periodization can take different forms including reverse periodization, where in contrast to traditional periodization, high-intensity low-volume training predominates during the preparatory period, before the volume is increased slightly, and intensity is maintained as the season progresses. Coaches and researchers have reversed the traditional order of volume and intensity (and therefore programming) of phases to yield different physiological and performance outcomes, sometimes subtle, but nevertheless different to traditional models [11]. Reverse periodization has received attention in both the coaching and scientific literature, especially in swimming [26, 27], and other endurance-oriented sports such as athletics or triathlon [28, 29]. Incorporating a higher proportion of high-intensity training early in the season is thought to stimulate physiological and performance adaptations. Reverse periodization has been used in combination with a polarized intensity distribution for improving sprint events in swimming [30]. However, a small number of relevant studies in swimming have not reported any substantial differences between traditional and reverse periodization models in enhancing 50-m performance, with a modest improvement of 1% in 100-m performance in both forms [27, 31]. A *polarized* three zone model of training is another approach characterized by covering ~ 80% of the volume in zone 1 (blood lactate [La^−^]b ≤ 2 mmol L^−1^) with most of the remaining 20% conducted in zone 3 (above velocity of 4 mmol L^−1^) [32, 33]. Reverse periodization has been evaluated in youth swimmers [26, 34], moderately trained runners [28, 35], recreational triathletes [29] and female fitness athletes [36].

All periodized models (traditional, blocks and reverse) can be considered a useful means of coordinating training to improve human sporting performance. However, more research is needed to provide a better understanding of the benefits of reverse training periodization in comparison with other models. The aim of this study was to conduct a systematic review of periodization studies to evaluate the effectiveness and utility of reverse periodization, and the influence of training volume/intensity in enhancing sports performance.

## Methods

### Search Strategy

A literature search was completed in December 2021 by two independent researchers (VR-C and JM-G) using the three industry-standard databases with no date restrictions: PubMed, Web of Science and Scopus. The search strategy consisted of identifying the relevant studies, with all terms searched in the title, abstract and keywords (where applicable).This systematic review was conducted following the guidelines of the Preferred Reporting Items for Systematic reviews and Meta-Analyses (PRISMA) statement [37].

The keywords used in the searches were: periodization, training, reverse, linear traditional and block. Title, abstract and keyword fields were searched using the following search strategy: ((("periodization" OR "Training") AND "reverse") AND ("linear" OR "traditional" OR "block")).

Following the literature search, the identification, screening, eligibility assessments and inclusion of studies were performed by the same researchers with disagreement settled by consensus. All duplicate references were removed, and remaining records examined by title and abstract to exclude irrelevant records. Studies were then selected following the eligibility criteria (Table 1). Descriptive data including publication details, modality, participant characteristics, study design, description of methods and results, were extracted from all eligible studies. If insufficient information was reported for any particular study, the authors were contacted to confirm the relevant details required.

**Table 1:** Summary of eligibility criteria of studies comparing reverse linear periodization with traditional and block periodization training approach for recreational and trained athletes

Criterion	Description
Type of participant	Healthy adult and younger distance runners, swimmers and triathletes
Type of intervention	
Methodology	Effects of reverse periodization vs other type of periodization training
Training intervention	At least 8 weeks
Type of outcome measure	
Periodization	Training zones, volumes and/or periodization details
Type of outcome	At least performance, physiological and anthropometric variables were evaluated
Type of study	Experimental design
Publication status	Peer-reviewed journal publication
Publication date	Publication date did not form part of the eligibility criteria
Language of publication	English language publication

### Inclusion Criteria

The summary of eligibility criteria is shown in Table 1. Studies were deemed eligible for further analysis if the following inclusion criteria were met: (1) when published in English language, (2) published in a peer-reviewed journal, (3) analyzed the effects of reverse periodization vs other type of periodization model, (4) involved at least 8 weeks of training intervention/analysis, (5) provided training zones, volumes and/or periodization details and (6) involved participants without a current injury or disability.

### Type of Participants

The level of the sample was classified as recreational and trained athletes using the criteria of each study included in the systematic review.

### Data Extraction

**Fig. 1:**
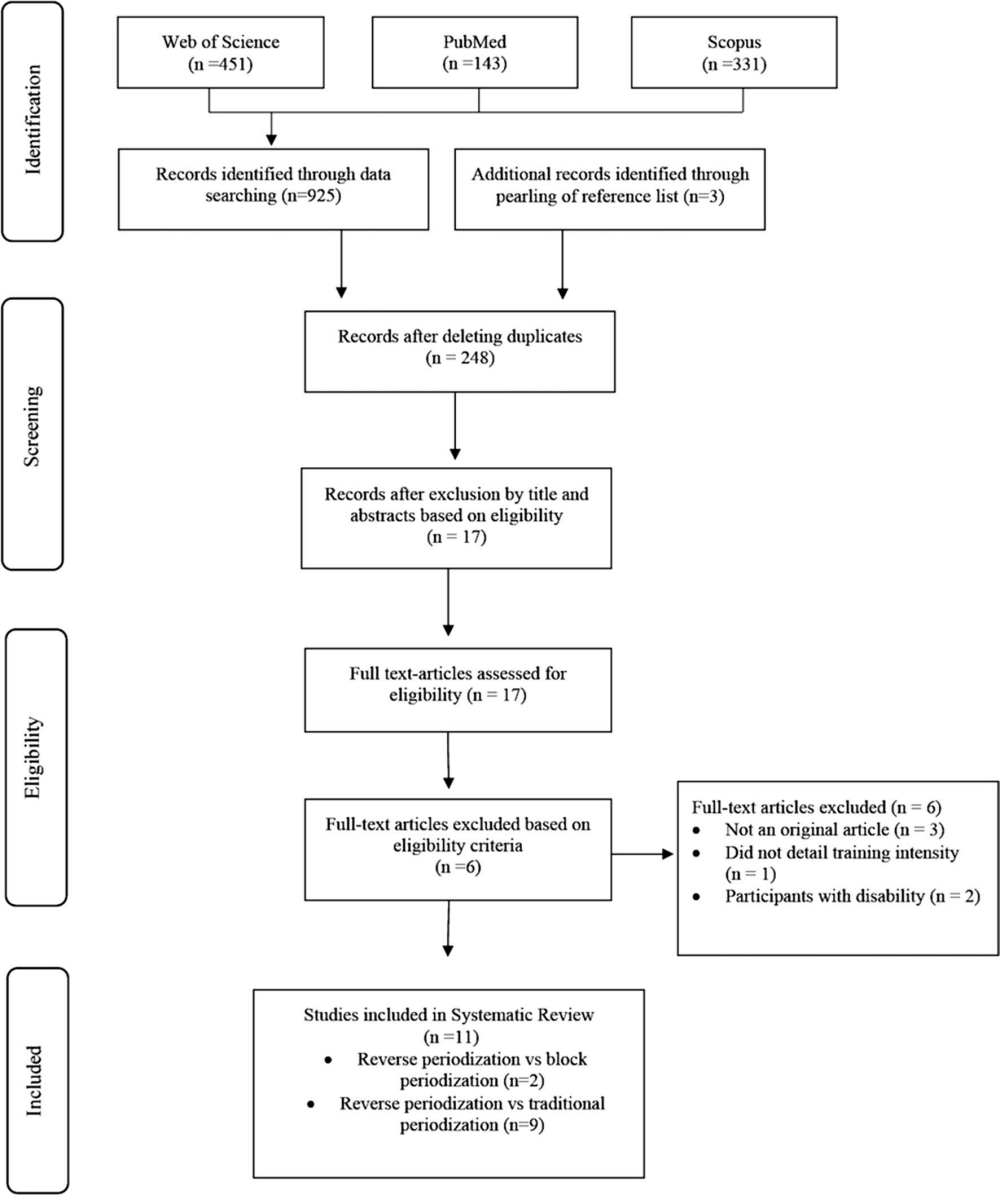
PRISMA flow diagram of the process used in selection of the journal articles included in the systematic review with the content of this article

Two of the authors (VR-C and JG-R) independently extracted characteristics of training protocols and results using a standardized form. A total of 11 studies were identified (Fig. 1).

### Quality Assessment

Two independent reviewers (VR-C and FG-M) analyzed the quality of included studies using the modified PEDro scale [38] and Oxford Levels of Evidence [39] (Table 2). The classic PEDro scale consists of 11 items to assess scientific rigor. A score of ≥ 6 represents the threshold for studies with a low risk of bias [40]. Item 1 is rated as Yes/No, while Items 2–11 are scored as 0 (absent) or 1 (present), and a score out of 10 is obtained by summation. Given that the assessors are rarely blinded, and that it is impossible to blind the participants and investigators in supervised exercise interventions for elite athletes, the items related to blinding (5–7) were removed from the scale for the purpose of this review. The maximum result on the modified PEDro 8-point scale was 7, as the first item was not included in the total score, resulting in a maximum score of 7 instead of 10, with adjusted quality ratings ranging from 6 to 7 deemed “excellent”, 5 “good”, 4 “moderate” and 0–3 “poor” [38]. Oxford Level of Evidence [39] scores range from 1a to 5, with 1a a systematic review of high-quality randomized controlled trials, and 5 an expert opinion.

**Table 2:** PEDro ratings and Level of Evidence of the included studies

Study	PEDro ratings									Oxford Level of Evidence
	1	2	3	4	5	6	7	8	Total	
Arroyo-Toledo et al. [26]	Yes	0	0	1	1	0	1	1	4	2b
Clemente-Suarez et al. [27]	No	1	0	1	1	0	1	1	5	1b
Bradbury et al. [28]	Yes	1	1	1	1	0	1	1	6	2b
Clemente-Suarez et al. [29]	Yes	1	1	1	1	0	1	1	6	2b
Clemente-Suarez et al. [31]	No	0	0	1	1	0	1	1	4	1b
Arroyo-Toledo et al. [34]	Yes	0	0	1	1	0	1	1	4	2b
Gómez Martín et al. [35]	Yes	1	0	1	1	0	1	1	5	2b
Prestes et al. [36]	Yes	1	1	1	1	0	1	1	6	2b
Clemente-Suarez et al. [41]	Yes	0	0	1	1	0	1	1	4	2b
Clemente-Suárez et al. [43]	No	1	0	1	1	0	1	1	5	2b
Rhea et al. [42]	Yes	1	0	1	1	0	1	1	5	2b

Items in the PEDro scale: 1 = eligibility criteria were specified; 2 = subjects were randomly allocated to groups; 3 = allocation was concealed; 4 = the groups were similar at baseline regarding the most important prognostic indicators; 5 = measures of 1 key outcome were obtained from 95% of subjects initially allocated to groups; 6 = all subjects for whom outcome measures were available received the treatment or control condition as allocated or, where this was not the case, data for at least 1 key outcome were analyzed by "intention to treat"; 7 = the results of between-group statistical comparison are reported for at least 1 key outcome; 8 = the study provides both point measures and measures of variability for at least 1 key outcome

## Results

### Final Study Selection

A total of 925 potential manuscripts were identified following database examination (Fig. 1). References list of selected manuscripts were also examined for any other potentially eligible manuscripts. Following this examination, 3 potential manuscripts were added. After removal of duplicates and elimination of papers based on title and abstract screening, 17 studies remained. Only 11 out of 17 studies met the inclusion criteria and were, therefore, included in the systematic review (Fig. 1).

### Characteristics of the Studies Selected

In terms of the quality of the studies selected, all studies were evaluated with the PEDro scale, with a mean score of 4.91 (Table 2). Using the Oxford Level of Evidence, two studies [27, 31] were classified as 1b (independent randomized controlled trial), while the remaining studies [26, 28, 29, 34–36, 41–43] were deemed as 2b (individual cohort study) level. The characteristics of the studies selected are presented in Table 3. A total of 11 intervention studies met all the inclusion requirements. Five studies performed reverse periodization in swimming [26, 27, 31, 34, 41], two studies in strength training [36, 42, 43], three studies in running [28, 35, 43] and one in triathlon [29]. Two of the studies compared block periodization and reverse periodization models [26, 35], whereas 9 studies compared traditional periodization and reverse periodization models [27–29, 31, 34, 36, 41–43].

**Table 3:** Characteristics of studies comparing reverse, traditional and block modifie periodization in trained athletes on physiological and performance measures

Study	Modality	Type of periodization	*n* (M/F) Age (years)	Experience	Training characteristics				Results
					Duration	Volume	Intensity	Measures	
Gómez Martín et al. [35]	Running	BP	8 (4/4) 37.2 ± 5.7	More than 6 years of experience on running training; competing at regional and national level in 10 km and half-marathon races	12 weeks	55 h and 20 min	60% in Z1, 23% in Z2 and 16% in Z3	Blood sample collection, running-based anaerobic sprint test, incremental test to exhaustion on a treadmill, countermovement jump and 10,000-m running performance	RP increased VO_2_max, speed at VO_2_max, heart rate at VT_2_ and VT_1_ and anaerobic performance in a running-based anaerobic sprint test, while BP improved VO_2_max, speed at VO_2_max and heart rate at VT_2_. Both types of training periodization maintained hematological values and evidently improved jump performance
		RP	8 (4/4) 37.0 ± 9.2			3246 min	60% in Z1, 23% in Z2 and 18% Z 3		
Bradbury et al. [28]	Running	TP	11 (8/3) 25.2 ± 7.4	More than 2-year running experience and a 5000-m personal best less than 25 min	12 weeks	295,090 m	–	Anthropometric measurements (body mass and 8 skinfolds), treadmill tests for running economy and VO_2_max, and a 5000-m time trial performance	TP and RP improved performance in 5000 m compared to the CG. No significant differences between the TP and RP. Similar improvements in VO_2_peak and Running Economy at 9 km/h and 11 km/h between TP and RP
		RP	11 (8/3) 25.2 ± 7.4			302,700 m	–		
		CG	13 (10/3) 28.2 ± 9.6						
Clemente-Suárez et al. [43]	Running	TP	30 (20/10) 25.5 ± 3.7	Amateur triathletes	8 weeks	2,741 TRIMPS		Motivation scale, adherence of training and 2000-m time trial performance	None of group modified their running performance. RP produced a decrease in heart rate, while TP and FT maintained heart rate. The basal HR presented significant differences between free training and reverse and traditional training groups. RP showed a significantly higher motivation with training than TP and FT. Regarding adherence to the training programs, there were no significant differences between groups
		RP				2,740 TRIMPS			
		FT				1610 TRIMPS			
Clemente-Suárez and Ramos-Campo [29]	Triathlon	TP	13 28.2 ± 9.6	More than 1 year of experience on triathlon training; 7.0 ± 1.5 h of training/week; competing at national level	10 weeks	37,754 TRIMPS		Body composition, heart rate variability, swimming, maximal horizontal jump and running performance and blood lactate concentration	RP and TP was an effective strategy to improve running performance, physiological variables, swimming technical ability, aerobic and anaerobic swimming performance, but did not modify body composition. RP efficiently improves horizontal jump performance compared with TP
		RP	11 25.6 ± 6.8			37,693TRIMPS			
		CG	8 25.9 ± 3.4			11,496 TRIMPS			
	Swimming							Autonomic response (heart rate variability) and 50 m swimming performance	
Clemente-Suárez et al. [31]		TP	7(4/3) 17.9 ± 1.9	6.5 ± 4.9 years of training experience and all of them competed at the national level at the time of the intervention	10 weeks	337,000 m	40% in Z1, 11% in Z2 and 48% in Z3		None of the groups improved their performance in the 50-m test. However, both groups exhibited changes in heart rate variability
		RP	10 (5/5) 17.5 ± 3.2			159,000 m	35% in Z1, 32% in Z2 and 33% in Z3		
Arroyo-Toledo et al. [26]	Swimming	BP	10 (0/10) 16.3 ± 1.1	Between 4 and 6 years of previous experience in swimming training and with practicing not more than three sessions per week and with moderately trained levels of competition	10 weeks	90,000 m	60% in Z1, 31% in Z2 and 9% in Z3	Body Composition and 100 m crawl swimming performance	RP improved 100 m swimming performance. BP increased fat-free mass while reducing values in fat mass and body fat percentage
		RP	10 (0/10) 15.6 ± 1.0			90,000 m	60% in Z1, 31% in Z2 and 9% in Z3		
Arroyo-Toledo et al. [34]	Swimming	TP	13 (7/5) 16.02 ± 0.6	Regional competitive program with average 5 years of training for a competition	14 weeks	324,000 m	70% in Z1, 25% in Z2 and 4% in Z3	100 m swimming performance and stroke rate, distance per stroke, specific swim power and maximal drag charge	RP improved 100 m swimming performance, specific swim power and maximal drag charge compared with TP values
		RP	13 (7/5) 16.02 ± 0.6			212,000 m	49% in Z1, 33% in Z2 and 17.90% in Z3		
Clemente-Suárez et al. [27]	Swimming	TP	7 (3/4) 17.9 ± 1.9	6.5 ± 4.9 years of training experience, training 5 to 6 days per week and all of them competed at national level	10 weeks	337,100 m	87% in Z1, 2.5% in Z2 and 10% in Z3	Velocity eliciting the blood lactate of 4 mmol/l, maximal oxygen uptake, rate of perceived exertion, heart rate, blood lactate concentration, strokes	Stroke index increased and stroke rate and RLPE at vVO_2_max decreased in TP. RP increased the VO_2_max
		RP	10 (5/5) 17.5 ± 3.2			159,000 m	841% in Z1, 7.9% in Z2 and 8% in Z3		
Clemente-Suárez et al. [41]	Swimming	TP	7 (3/4) 17.9 ± 1.9	6.5 ± 4.9 years of training experience and all competing at the national level	10 weeks	337,050 m	87% in Z1, 2.5% in Z2 and 10% in Z3	Swimming velocity, energy cost and percentage of aerobic and anaerobic energy contribution to the swimming intensities corresponding to the aerobic threshold, the anaerobic threshold and the velocity at maximal oxygen uptake	Both groups increased % anaerobic energy. In contrast, at the anaerobic threshold intensity and energy cost were only increased in TP. The percentage of aerobic, anaerobic, energy expenditure, energy cost at vVO_2_max and swimming velocity did not alter in both groups
		RP	10 (5/5) 17.5 ± 3.2			159,020 m	84% in Z1, 7.9% in Z2 and 8.1% in Z3		
Prestes et al. [36]	Strength training	TP	10 (0/10) 27.6 ± 1.15	More than 6 months of previous experience with strength training	12 weeks	9,477 total repetitions	5.4% in < 6 RM, 67% in 7–11 RM and 27% in > 12 reps	Body composition (fat mass and fat-free mass), maximal strength (bench press, lat pull-down, arm curl and leg extension) and local muscular endurance	TP increased fat-free mass and decreased fat mass. Both models yielded gains in maximum strength levels in all exercises analyzed – higher in TP
		RP	10 (0/10) 26.2 ± 0.92			9,484 total repetitions	5.4% in < 6 RM, 68% in 7–11 RM and 27% in > 12 reps		
Rhea et al. [42]	Strength training	TP	20 (10/10) 21 ± 2.2	Subjects from college weight-training courses with more than one year and a maximum of 5 years training experience	15 weeks	85,500 ± 23,500 kg	–	Muscular endurance, total strength (1RM) and leg circumference	No differences in endurance, strength and leg circumference gains between groups. RP was more effective than TP at increasing muscular endurance. DUP and TP achieved higher increases in strength than RP
		RP	20 (10/10) 22 ± 1.6			82 150 ± 28,600 kg	–		
		DUP	20 (10/10) 21 ± 1.9			80,120 ± 28,820 kg			

Data are mean ± SD; RP reverse periodization, TP traditional periodization, BP block periodization, CG control group, FT free training, DUP daily undulating periodized, vVO2max: velocity at Maximum oxygen consumption

*Z1* Zone 1, *Z2* Zone 2, *Z3* Zone 3

Six studies were conducted in mostly recreational athletes and five in trained athletes. There were a total of 230 athletes in the included studies, involving a total of 134 females (58%). The mean age of the athletes was 23 y (standard deviation of 6 y), with a range of 16–37 y. Two of the studies assessed females only, nine studies involved both males and females, and none of the studies assessed males only. In addition, only two studies used a control group to evaluate periodization models during the experimental intervention. The training programs evaluated in this review were predominantly short-term interventions [26, 27, 29, 31, 41, 43] lasting ~ 10 weeks, and only four studies had a duration equal to or greater than 12 weeks [28, 34–36]. The mean duration of the training interventions was 11.5 ± 1. 5 weeks. One of the studies was 8 weeks, five were 10 weeks, three were 12 weeks, one was 14 weeks and one was of 15 weeks' duration. All studies except that of Clemente-Suárez and Ramos-Campo [29] provided quantitative details of the training volume, and all studies except that of Rhea et al. [42] and Bradbury et al. [28] provided the training intensity of the training intervention. In addition, the study of Clemente-Suárez and Ramos-Campo [29] and Clemente-Suarez et al. [43] provided the training load in training impulse (TRIMPS) units.

**Fig. 2:**
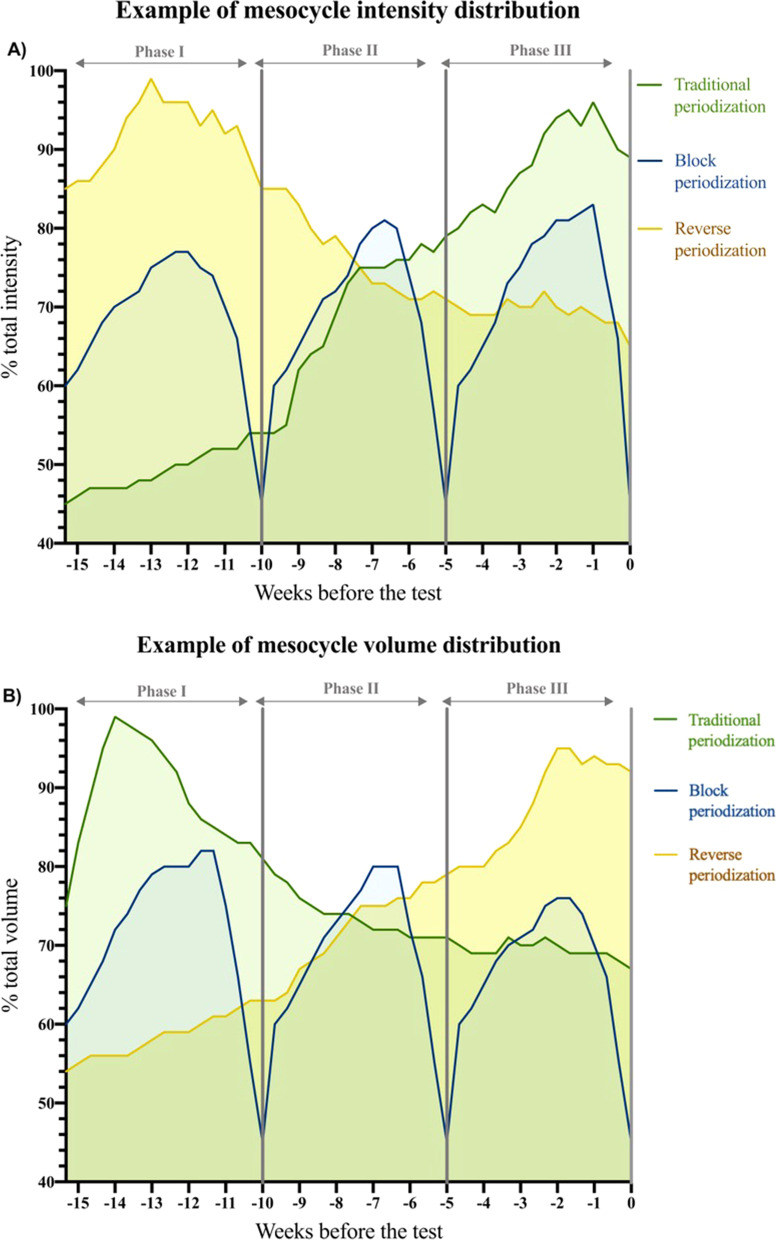
Example of mesocycle distribution of traditional periodization, block periodization and reverse linear periodization. **A** Intensity distribution of the different periodization models. **B** Volume distribution of the different periodization models

Three typical patterns detailing the distribution of training intensity in a macrocycle - traditional periodization, block periodization and reverse periodization - are illustrated in Fig. 2. Training intensity distribution (TID) was shown only in six studies. Traditional periodization was characterized by programming that used a pyramidal TID (characterized by a decreasing training volume in zones 1, 2 and 3 [80%) of the volume is conducted in z1, and the remaining 20% in Z2 and Z3]) in the studies of Arroyo-Toledo et al. [34] and polarized TID (characterized by covering ∼80% of the volume at Z1, with most of the remaining 20% conducted at Z3) in the studies of Clemente-Suárez et al. [27, 41] and Clemente-Suárez and Ramos-Campo [29]. The reverse periodization was featured as a polarized TID in the studies of Clemente-Suárez et al. [27, 31, 41], and pyramidal TID in the studies of Arroyo-Toledo et al. [26, 34]. Gómez Martin et al. [35] used a polarized TID in the first mesocycle, and a pyramidal distribution in the second and third mesocycle for the reverse periodization group, while the block periodization applied a polarized distribution in the second mesocycle and a pyramidal distribution in the first and third mesocycles. Block periodization using a pyramidal TID was employed in the studies of Arroyo-Toledo et al. [26] and Gómez Martin et al. [35]. In relation to the strength training studies, the recreationally trained women of the study of Prestes et al. [36] performed 67% of training between 7 and 11 repetition maximum (RM), followed by 27% of training > 12RM, and 5% < 6RM. This classification was used in the review following the guidelines established by Haff et al. [44]. However, Rhea et al. [42] did not report the training intensity used for the periodization groups.

Regarding training volume, the running studies reported the volume using different metrics of either time or distance. The athletes in the study of Gómez Martin et al. [35] performed about 3300 min of training over 12 weeks, without substantial differences between periodization model groups. In the case of the study of Bradbury et al. [28], the runners completed 290–300 km in 12 weeks without substantial differences in the mean weekly volume between the periodization groups. However, this volume differed between the training blocks according to the periodization model. All swimming studies displayed the training volume in meters. In the studies of Clemente-Suárez et al. [27, 31, 41] conducted with the same sample of athletes, those swimmers undertaking traditional periodization performed double the training volume of the reverse periodization swimmers (340 km *vs.* 160 km). In addition, the traditional periodization group performed 324 km compared to 212 km for the reverse periodization group in the study of Arroyo-Toledo et al. [34]. However, the same training volume was performed by the block and reverse periodization groups (90 km) in the study of Arroyo-Toledo et al. [26]. Finally, regarding the strength training studies, the athletes of Prestes et al. [36] performed a total of 9,500 repetitions without a substantial difference between periodization model groups. Similarly, the athletes in the study of Rhea et al. [42] lifted between 80,000 and 85,000 kg without differences between periodization model groups.

### Effects on Physiology Parameters

There are three main physiological parameters [45] affecting endurance performance: (i) maximal oxygen uptake (V̇O_2max_), (ii) lactate threshold and (iii) movement economy. Both reverse periodization and block periodization training have yielded similar improvements in V̇O_2max_ and the velocity corresponding to V̇O_2max_ (vV̇O_2max_) [35]. Greater improvements in V̇O_2max_ for reverse periodization and reductions for the traditional periodization model were reported in the study of Clemente-Suárez et al. [27]. Similar improvements in running economy and peak oxygen uptake (V̇O_2peak_) were reported for traditional and reverse periodization [28]. Energy cost of swimming was impaired following traditional periodization, without any substantial changes after reverse periodization [41]. Finally, aerobic and anaerobic thresholds remained largely unchanged following both traditional and reverse periodization [41].

### Effects of Exercise Performance

Two studies [27, 31] reported 50-m swimming performance with reverse periodization compared to traditional periodization. The pre-post training intervention times in the 50-m test were similar with both forms of training (traditional periodization: 28.81 ± 1.72 vs. 28.78 ± 1.44 s; reverse periodization: 29.50 ± 2.07 vs. 30.24 ± 2.83 s). The studies of Arroyo-Toledo et al. [26, 34] reported an improvement of 100-m swimming performance in both forms of periodization (5% in 100-m time in reverse periodization and 1.2% in block periodization). In relation to running performance, 2000 m [29] and 5000 m [28] time trials improved 2.4% after 12 weeks of both reverse periodization and traditional periodization training. In the case of the study of Clemente-Suárez et al. [43], the authors did not find improvements in the performance of 1000 m running test regarding the use of traditional or reverse periodization. Similarly, both forms of periodization showed gains in maximum strength levels (1RM) with different exercises analyzed in the study of Prestes et al. [36]. However, the increases were greater with traditional periodization when compared with reverse periodization. Regarding muscular endurance gains, both forms of periodization increased similarly (16 and 15% for reverse periodization and traditional periodization respectively)[42].

## Discussion

This systematic review identified 11 studies that directly compared traditional periodization (*n* = 9) and block periodization (*n* = 2) training with reverse periodization. Studies were conducted in both recreational [28, 29, 31, 35, 42, 43] and trained athletes [26, 27, 34, 36, 41]. The training programs evaluated in this review were predominantly short-term interventions [26, 27, 29, 31, 41, 43] lasting ~ 10 weeks, and only four studies had a duration longer than 12 weeks [28, 34–36] ranging from 12 to 15 weeks. The short duration of the interventions in periodization studies makes it difficult to draw firm conclusions regarding longer-term changes in exercise and/or sports performance of any particular periodization model.

In relation to competitive (sports) performance, 5 of the 11 studies included in this review were in swimming. A systematic review on swimming periodization identified that the traditional periodization was the most common form used in well-trained swimmers, but only four studies compared traditional versus reverse periodization [5]. Our results suggest that reverse periodization improved swimming performance [26] more than block periodization, while Clemente-Suárez and Ramos-Campo [29], reported a similar improvement in swimming technical ability and swimming performance with reverse periodization and traditional periodization. However, neither traditional (characterized by pyramidal TID) nor reverse periodization (characterized by polarized TID) yielded significant improvements in 50-m swimming performance [27, 31]. Only two studies [26, 34] reported significant improvements in 100-m swimming performance following reverse periodization and block periodization. The greater improvements for the reverse periodization group (5%) could be explained by the low performance level of swimmers used in these studies (~ 65 s in 100-m), or a greater specificity of stimulus in the first weeks of training (high-intensity training). In addition, it appears that traditional periodization can improve the swimming efficiency by ~ 2% most likely related to the higher volume of technical work performed during the training program, while reverse periodization can increase the VO_2max_ by 6.4% in trained swimmers [27]. Reverse periodization has been used in combination with a polarized TID for improving performance in sprint events. On the other hand, both reverse periodization and traditional periodization improved 2000 m and 5000 m running time trials [28, 43], without a substantial difference between periodization models, and anaerobic running performance improved in reverse periodization compared to block periodization; although the sample was recreational runners, the study supports the proposition that both periodization models are better than non-planned training [35]. However, the study of Clemente-Suárez et al. [43] did not show improvements in 1000 m performance regarding the use of traditional or reverse periodization. These results indicate that reverse periodization could be a viable alternative for improving performance in short distance events (primarily anaerobic in nature) such as the 100-m swim event, while traditional periodization seems to be the best choice for long distance (swimming) events, without a clear effect on short sprint events such as the 50-m swim or middle and long-distance running events. The lack of effects on swim performance could relate to training a variety of fitness adaptations rather than emphasizing the primary fitness characteristic [11].

To our knowledge, only two studies have reported greater gains in 1RM strength in traditional periodization/programming as opposed to reverse periodization/programming [36, 42]. Regarding the effects of periodization on muscular strength, Prestes et al. [36], reported increases in muscular strength for both forms of periodization (traditional periodization *vs.* reverse periodization) in bench press (17% and 16%), lat pull-down (30% and 22%), arm curl (20% and 16%) and leg extension (37% and 32%). However, Prestes et al. [36], asserted that traditional periodization rather than reverse periodization is more effective for strength and hypertrophy. There is a possibility for traditional periodization to be more effective as it allows for more quality training with heavier weights at the end of the program [36]. A similar comparison also showed a greater increase in strength after traditional periodization in the study of Rhea et al. [42]. However, both reverse periodization (16%) and traditional periodization (15%) showed a similar increase in muscular endurance [42]. Analysis of the effect size (ES) indicates that traditional periodization was more effective at eliciting strength than reverse periodization [42] (ES = − 0.31). Both studies matched the intensity and volume of training, with the only difference being the distribution of training over the weeks. The similar increase in muscular strength for both periodization approaches likely relates to the training stimulus involving matched loads, and a similar pattern of the functional responses to training stress. With respect to improvements in muscular endurance, reverse periodization was characterized by decreased intensity and increased volume toward the last few weeks of training in these studies, which is more like a strength-endurance training stimulus. It seems reasonable to improve the muscular endurance with training more specific to this strength attribute before the post-test evaluation. Prior training history will influence adaptations to further training interventions, particularly in strength training [46]. Although subjects are typically categorized as recreational or trained, only the study of Prestes et al. [36] formally detailed that the subjects performed at least three times per week (3 × 10RM) in the previous 6 months, without details of the periodization model used. Similarly, the study of Rhea et al. [42] only reported that subjects participated in strength training programs for at least 12 months, but without specifying the underlying training and periodization model.

In addition to effects on performance and physiological parameters, different types of periodization may have variable effects on body composition. Arroyo Toledo et al. [26] reported that block periodization can elicit more favorable improvements in body composition than reverse periodization in moderately trained female swimmers. The primary premise of block periodization is employing highly concentrated training workload phases (periodization blocks) to stimulate adaptation and residual effects [26]. The blocks must be sequenced in a logical order to benefit from the residual effects [26]. Reductions in fat mass can be achieved during a period of high-intensity training [46], and including a specific phase of training for this purpose maybe useful in sports where body composition is important for performance.

There were some limitations to this review given the heterogeneity of sports, training and methodological approaches of the underlying studies. There was substantial inter-individual variability regarding the participants in the different studies (which included teenage swimmers, local/regional swimmers, experienced runners, etc.) across all performance variables that may have impaired the ability to establish conclusive outcomes in this systematic review. In addition, as periodization generally refers to periods of a season or more, it may be logical for future research to evaluate longer periods, so that differences after each periodization model can become more pronounced. A critical drawback in some of these studies is the lack of a randomized controlled design (the majority of studies did not equalize volume nor intensity when comparing two different workloads across time) as shown in Table 3. For example, the total volume of traditional periodization during 10 weeks of training in one study was more than 337,000 m, while for the reverse periodization the volume was only ~ 160,000 m [27, 31]. The absence of a control group did not reflect the improvements in periodized models vs. control group. More research over a longer term is needed to develop a stronger evidence base comparing and contrasting the different types of periodization models. Most of the existing studies have not reported details of nutritional status, fatigue levels and/or variations in motivation and other psychological attributes, that can all influence adaptation and performance. Future work will identify individual athlete characteristics associated with the different models of periodization, and which events and sports might benefit substantially from reverse periodization training.

## Conclusion

It is not clear if reverse periodization is more effective in improving sports performance than other periodized models. Use of reverse periodization likely induces similar improvements to a traditional model in shorter events such as the 100-m swimming event. Comparative studies of periodization models in endurance sports require careful planning of experimental design, longer study periods, and where appropriate careful matching of training volumes and intensities.

## Data Availability

All data and material reported in this systematic review are from peer-reviewed publications.

## Abbreviations

[La-]b: Blood lactate
TRIMP: Training impulse
TID: Training intensity distribution
RP: Reverse periodization
RM: Repetition maximum
V̇O2max: Maximal oxygen uptake
V̇O2peak: Peak oxygen uptake
ES: Effect size
